# A Deep Dive Into the Newest Avenues of Immunotherapy for Pediatric Osteosarcoma: A Systematic Review

**DOI:** 10.7759/cureus.18349

**Published:** 2021-09-28

**Authors:** Megha Suri, Nitin Soni, Nkiruka Okpaleke, Shikha Yadav, Suchitra Shah, Zafar Iqbal, Mohammed G Alharbi, Harjeevan S Kalra, Pousette Hamid

**Affiliations:** 1 Medicine-Pediatrics, California Institute of Behavioral Neurosciences & Psychology, Fairfield, USA; 2 Medicine, California Institute of Behavioral Neurosciences & Psychology, Fairfield, USA; 3 Psychiatry and Behavioral Sciences, California Institute of Behavioral Neurosciences & Psychology, Fairfield, USA; 4 Internal Medicine, California Institute of Behavioral Neurosciences & Psychology, Fairfield, USA; 5 Emergency Medicine, California Institute of Behavioral Neurosciences & Psychology, Fairfield, USA; 6 Internal Medicine/Emergency Medicine/Oncology, California Institute of Behavioral Neurosciences & Psychology, Fairfield, USA; 7 Neurology, California Institute of Behavioral Neurosciences & Psychology, Fairfield, USA

**Keywords:** tumor immune microenvironment, immune checkpoint therapy, osteosarcoma, immunotherapy, car t, nk cell, immunoediting, mesenchymal stem cells (mscs)

## Abstract

Osteosarcoma (OS) is the most common primary bone cancer affecting children and young adults, most often occurring at the metaphysis of long bones. At present, treatment with combinations of surgery and chemotherapy for the localized OS has only brought minuscule improvements in prognosis. In comparison, the advanced, metastatic, or recurrent forms of OS are often non-responsive to chemotherapy, adding to the dire need to develop new and efficient therapies.

The question of interest investigated in this systematic review is whether immunotherapy can play a meaningful role in improving the clinical outcomes of children with OS. This article aims to summarize the preclinical and clinical research conducted thus far on potential therapeutic avenues for pediatric OS using immunotherapy, including methods like checkpoint inhibition, adoptive cellular therapy with T-cells, chimeric antigen receptor T (CAR-T), and natural killer (NK) cells. It also highlights the influence of the innate and adaptive immune system on the tumor microenvironment, allowing for OS progression and metastasis.

This systematic review contains 27 articles and analyses of multiple clinical trials employing immunotherapeutic drugs to 785 osteosarcoma participants and over 243 pediatric patients. The articles were obtained through PubMed, PubMed Central, and ClinicalTrials.gov and individually assessed for quality using the Assessment of Multiple Systematic Reviews (AMSTAR) checklist and the Cochrane risk-of-bias tool. The reviews reveal that immunotherapy's most significant impact on pediatric OS includes combining immune checkpoint blockers with traditional chemotherapy and surgery. However, due to the bimodal distribution of this aggressive malignancy, these studies cannot precisely estimate the overall effect and any potential life-threatening adverse events following therapy in children. Further research is required to fully assess the impact of these immunotherapies, including more extensive multinational clinical trials to focus on the pediatric population.

## Introduction and background

Osteosarcoma (OS), also known as osteogenic sarcoma, is one of the most commonly encountered bone malignancies worldwide, occurring in 5% of children globally [1]. Before implementing chemotherapy, the outcome of patients with OS was poor, with a survival rate of less than 20% just before the 1970s. Shortly after introducing surgical resection with adequate margins and combinations of double or triple chemotherapy (i.e., cyclophosphamide and etoposide, gemcitabine and docetaxel, or high-dose methotrexate, etoposide, and ifosfamide) [2], the survival rate increased immensely [3]. However, despite surgery and cytotoxic therapy, approximately 30% of patients relapse within five years, with lung and bone metastases being the most prevalent sites of recurrence [4,5]. Patients with advanced, metastatic, and recurrent OS continue to experience quite a poor prognosis. Overall, the survival rate is less than 20% [3,6]. The values reflect a stagnant survival rate due to the lack of new treatment strategies, especially in the frontier of pediatric OS [7]. The rarity, heterogeneity, and difficulty of detecting a tumor-specific antigen are the critical reasons for the lack of advancement in this population [8]. As a result, novel treatments are needed in urgency to improve the outcomes in children with cancer.

Compared to other childhood bone sarcomas, osteosarcoma's distinct pathological and clinical features continue to produce inadequate responses [7]. The aggressive tumor is highly thought to be derived from mesenchymal stem cells and is discovered mainly along the metaphysis of lower long bones in children and young adolescents. It includes several comparable histological subtypes with the unified hallmark, including osteoid-producing malignant cells [9]. The OS subtypes also carry many genetic mutations, which will provide tremendous potential for targeted therapy [10]. However, while considering the characteristics of this malignancy, more profound knowledge of the tumor microenvironment (TME), the roles of the innate and adaptive immune systems driving sarcoma progression, and the fundamentals of immunoediting is essential before targeted therapies may be implemented further for the treatment of OS.

This review will integrate targeted immunotherapy for pediatric osteosarcoma while summarizing the overall clinical research conducted thus far. Furthermore, potential avenues of therapy using mesenchymal stem cells (MSCs) and adoptive cell transfer (ACT) are discussed and analyzed with the hope of discovering any combinational strategies that may provide therapeutic benefit for children.

## Review

Methods

This systematic review is designed to report results applying the Preferred Reporting Items for Systematic Reviews and Meta-Analyses (PRISMA) guidelines [11].

Search Strategy

A systematic literature review was completed using databases from 14 May 2021 up to 21 July 2021. Eligible articles were explored thoroughly and identified by a search of PubMed and PubMed Central. The search strategy and Medical Subject Heading (MeSH) terms and keywords were employed to precisely filter relevant articles which demonstrate the use of immunotherapy for pediatric osteosarcoma. The keywords used include child, osteosarcoma, sarcoma, osteogenic sarcoma, immunotherapy, and cancer immune therapy. The Boolean scheme was implemented to the keywords and the MeSH strategy format to screen articles within PubMed. The search performed electronically included original studies on human subjects published in the English language. Furthermore, we searched ClinicalTrials.gov search for clinical trials involving immunotherapy and osteosarcoma in children, and two authors independently searched for additional citations extracted data from each eligible study.

Inclusion and Exclusion Criteria

The screening process to recognize all citations of potential acceptability was performed by two reviewers independently. From the articles obtained, we ensured that study participants included children under 18 years of age. We restricted our choice of studies to systematic reviews and meta-analyses of recognized abstracts and full texts that were applicable. We excluded clinical trials without results, location, or studies without author names. Articles related to animal studies were also excluded. For final eligibility, only papers published between the years 2016 and 2021 in the English language were included in the synthesis of this review.

Data Extraction

Data selection and extraction were obtained independently by three researchers (MS, SY, and SS) using a standardized recording tool to document the authors, study design, year of publication, country of origin, number of study participants and their characteristics, immunotherapy intervention, and the study outcomes.

Methodological Quality Assessment

The clinical trials were overseen using the Cochrane risk-of-bias tool and the Newcastle-Ottawa scale, while the systematic reviews were subjected to quality appraisal using Assessment of Multiple Systematic Reviews (AMSTAR). Each study was individually assessed with specific criteria and variables to disclose any areas of potential biases. Through this method, we were able to determine the intrinsic methodological quality of the research papers, with scores above 8 marking the point of reference for inclusion.

Results

**Figure 1:**
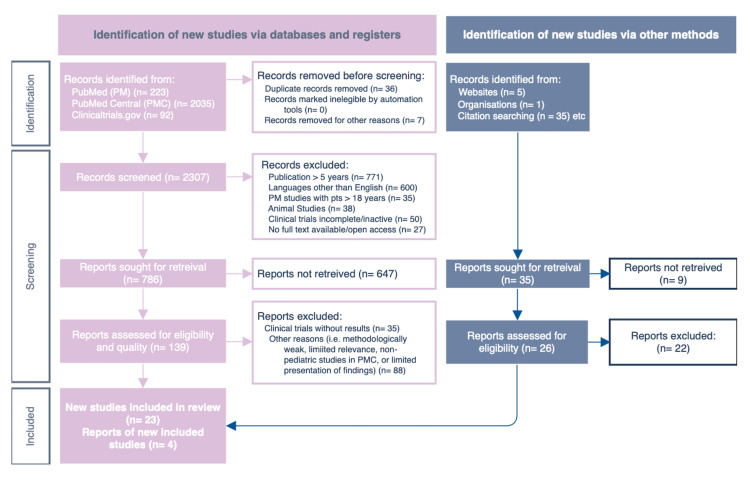
PRISMA flow chart. PRISMA: Preferred Reporting Items for Systematic Reviews and Meta-Analyses.

Our preliminary search resulted in a total of 2,391 articles. Among the 2,391 articles discovered, 223 were from PubMed, 2,035 studies were found in PubMed Central, 92 clinical studies were located through clinicaltrials.gov, and 41 papers were obtained via reference review. Of the total value, we excluded 43 articles by screening for duplicates and additionally removed 1,527 of them after screening for studies based on their eligibility to our inclusion criteria, matching for the age of participants, years of publication, studies performed in humans, completed or ongoing clinical trials, availability of full or open texts, and those published in the English language. The remainder of 821 articles were then filtered based on their respective title, including those with particular relevance to osteosarcoma and immunotherapy use in children. The final screening process included 165 articles, in which 138 were discarded due to the lack of results, methodologically weak studies, or reporting limited presentation and findings to our ongoing research. Overall, 27 articles were considered eligible for final analysis. Figure 1 illustrates the performed search according to the Preferred Reporting Items for Systematic Reviews and Meta-Analyses (PRISMA) below.

The studies, therapies, and literature examined for this article were all unique. However, the objectives were comparable. A structured summary of the reviewed literature is displayed below in Table 1.

**Table 1:** A tabulated summary of the study characteristics. Abbreviations – ACT: adoptive cellular transfer; ATCT: adoptive T-cell transfer; CTLA-4: cytotoxic T lymphocyte-associated protein 4; ICB: immune checkpoint blockade; MSCs: mesenchymal stem cells; OS: osteosarcoma; PD-1: programmed death cell receptor 1; TME: tumor microenvironment; YOP: year of publication.

Author	YOP	Journal	Country	Study type	Primary endpoints of the study
Wang et al. [12]	2016	Front Immunol	China	Review	T-cell-based treatments for OS, with a focus on combination methods to boost the efficacy of ATCT.
Lindsey et al. [7]	2017	Rheumatol Ther	USA	Review	Summarizes current knowledge of the genesis of OS, diagnostic techniques, and the current standard of treatment. Presents a range of experimental treatments, as well as data that support a prospective shift toward immunomodulation.
Rivera-Cruz et al. [13]	2017	Stem Cells	USA	Review	A discussion of the mechanisms by which MSCs are able to modulate the adaptive and innate immune responses, including the relationship between MSCs and immune cells within the TME.
Nathenson et al. [14]	2017	Oncologist	USA	Review	The author discusses the history of immunotherapy research in the treatment of soft tissue and bone sarcomas, as well as the current state of the field, with a focus on vaccination trials, ATCT, and immune checkpoint inhibition.
Grohar et al. [9]	2017	Am Soc of Clin Oncol	USA	Author manuscript	The clinical and demographic features in treatment for Ewing sarcoma and osteosarcoma, including the biology of the germline mutation.
Wedekind et al. [15]	2018	Paediatr Drugs	USA	Review	The current state of cancer immunotherapies, including effectiveness and toxicity in pediatric patients, as well as new prognostic biomarkers that might lead to individualized treatments.
Dyson et al. [10]	2019	J Hematology Oncol	USA	Review	Highlighting the TME and specific immunotherapeutic targets.
Miwa et al. [8]	2019	J Oncol	Japan	Review	The authors discuss immune surveillance for cancer, the history of immunotherapy, and the latest clinical studies on OS immunotherapy.
Jiang et al. [16]	2019	Cell Prolif	China	Review	Reviews the mechanisms regulating the immune modulation function of pro- and anti-inflammatory cells, with a focus on MSCs and their immunosuppressive effects.
Casey et al. [17]	2020	Cancer Immunol Res	USA	Author manuscript	Neoepitope expression and future advances of T-cell infiltration into the immunosuppressive TME.
Birdi et al. [18]	2020	J Immunother Cancer	Canada	Review	Highlights clinical data supporting how immunotherapy is being used in soft tissue sarcoma and bone sarcomas.
Zhang et al. [19]	2020	Medicine	China	Review and meta-analysis	The results of survival analysis of potential prognostic genes are significantly associated with childhood OS.
Clemente et al. [20]	2021	J Transl Med	Italy	Review	Gives insight on groundbreaking advances in the immune-therapeutic field, as well as the possible applications of immunological therapies in sarcomas, including ICB via modification of the axis in CTLA-4 and PD-1, plus therapies with ACT.
Dong et al. [21]	2021	Front Immunol	China	Review	Discusses immune cells in the TME and new immunotherapy strategies based on immune cell modulation.
Rathore et al. [2]	2021	J Clin Med	USA	Review	The biological mechanisms that contribute to tumor development are investigated, and this information is used to describe new therapy options for OS.
Gazouli et al. [22]	2021	Cancers	Greece	Review and meta-analysis	A detailed analysis of recurrent osteosarcoma treatment strategies over the last two decades. This report compares the current treatment strategies to the objective responses in potential therapies of preclinical and clinical trials.

Discussion

Over the last decade, significant advancements in outcomes have befallen various solid-tumor malignancies based on new research of the tumor microenvironment and its interaction with antitumor immune cells, the causes of tumor invasion, and isolation of tumor-specific antigens. However, the foundation of patient-specific treatments entails a comprehensive understanding of the tumor's biology and genetics. Below, we highlight the tumor genetics involved in osteosarcoma progression and the frontiers of immunotherapy employed for therapy in children.

Tumor microenvironment and the immune system

Cancer progression emerges from a complex interplay between the TME and the many cells involved in forming the matrix, including fibroblasts, endothelial cells, and immune cells [10,16]. The TME consists of a mesh between the innate (i.e., macrophages, neutrophils, monocytes, natural killer [NK] cells, and antigen-presenting cells like dendritic cells) and adaptive immune cells (i.e., B lymphocytes, CD4+ helper-T cells, and CD8+ cytotoxic-T cells) [8,10]. The adaptive immune response is acquired by individuals throughout their lifetime and is induced by specific immune responses, causing antibodies to a particular pathogen. This response is significantly different from the innate immune system, where previous exposures to pathogens are not responsible for immediate immunity [13]. When foreign antigens are detected, the interaction between innate and adaptive cells promotes immunosuppressive effects on the immune system, causing a release of cytokines to eliminate pathogens and remove damaged cells.

**Figure 2:**
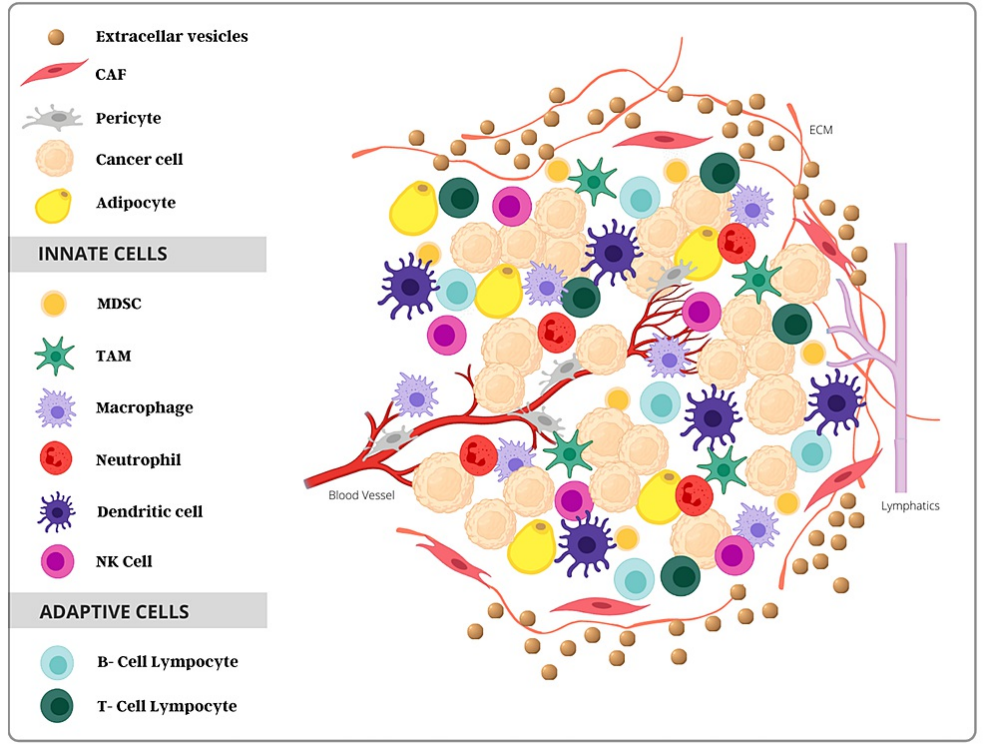
Tumor microenvironment consisting of immune cells from the innate and adaptive systems. Abbreviations – CAF: cancer-associated fibroblast; MDSC: myeloid-derived stem cell; TAM: tumor-associated macrophage; NK, natural killer.

Conversely, when the standard mechanism fails, the TME adjusts to block the immune system’s response and allow the tumor to “escape” the necessary inflammatory response. Additionally, the presence or absence of many specialized immune cells has also foreshadowed prognosis in pediatric osteosarcoma (OS) [23]. With an increased understanding of the sarcoma TME and the immunological markers that allow for tumor progression, the use of targeted immunotherapy and tumor modulation may have an overall significant clinical impact in treating children with OS. The cells of the innate and adaptive immune systems that comprise the tumor microenvironment are displayed in Figure 2 below.

Immunoediting

**Figure 3:**
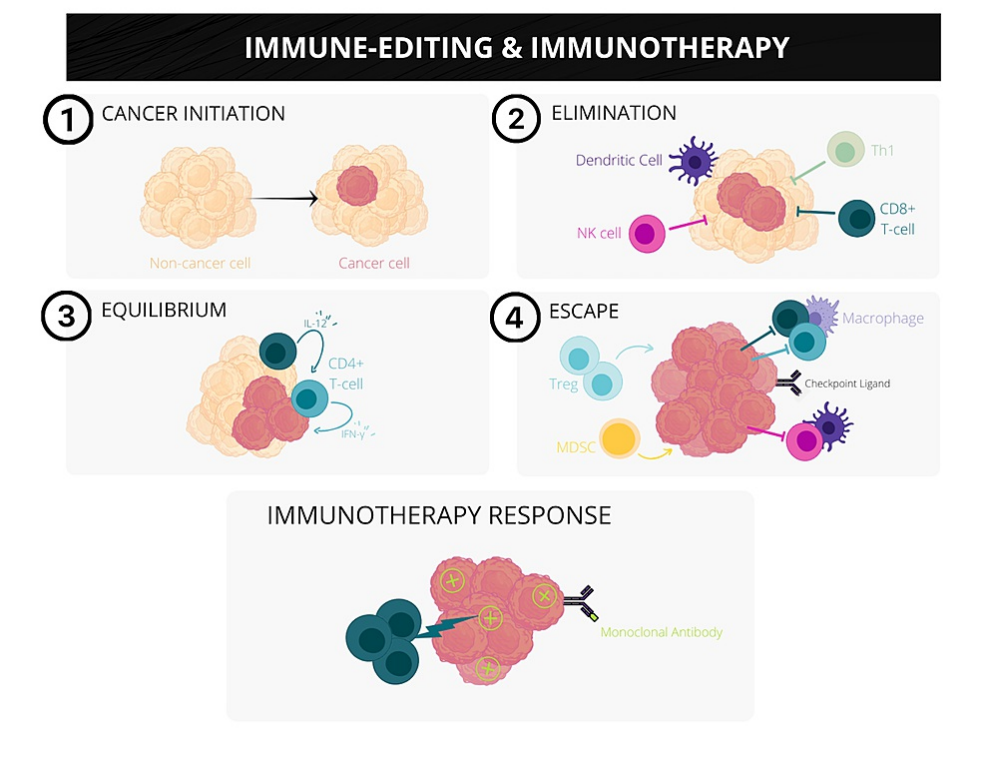
Three stages of immunoediting and cancer response to immunotherapy. The progression of cancer depends on three stages of immunoediting: (1) elimination, (2) equilibrium, and (3) escape. As cancer cells remain undetectable during the initial two stages, the over-expression of T-lymphocytes in the final stage induces immunosuppression. The illustration demonstrates the potential application of immunotherapy in its ability to overcome the tumor response and cause regression of cancerous cells. Abbreviations – NK: natural killer; Th1: type 1 helper T cell; IL-12: interleukin-12; IFN-γ: interferon-γ; MDSC: myeloid-derived suppressor cell.

Immunoediting is a dynamic process discovered by Schreiber et al. describing the transformation of normal healthy cells to clinically detectable cancer. The theory identifies three distinct stages: elimination, equilibrium, and escape [24]. The first stage of elimination, previously known as immunosurveillance, includes the combination of innate and adaptive cells destroying cancer cells before becoming detectable. However, some cancer cells may survive the initial intervention and forego the second stage. During equilibrium, the adaptive immune cells inhibit the growth of cancerous cells by editing the tumor immunogenicity, meaning some cells remain clinically silent [25]. The cancer cells not recognized by the host immune system avoid the attack response and become susceptible to enter the third and final stage. The immune system becomes significantly suppressed during the escape phase as more tumor cells continue to replicate [24]. In an attempt to control the newly replicated cancer cells, T-lymphocytes become overwhelmed and exhausted, ultimately leading to cancer progression. Immunotherapies aim to counteract this escape mechanism by targeting the TME and overcoming the patient’s immune system by recognizing and removing cancerous cells altogether. However, our potential for using this knowledge to develop cancer immunotherapies for children is still very much in its early stages. Figure 3 illustrates the stages of cancer progression as described above.

Mesenchymal stem cells and osteosarcoma

Mesenchymal stem cells (MSCs) represent a specific population of cells found within the tumor stroma, playing an integral role in promoting or inhibiting tumor growth. Known for their multipotent and self-renewal abilities in osteoblasts, chondrocytes, and adipocytes, MSCs represent a promising tool for osteosarcoma (OS) cell therapy, particularly for their antitumor effects from resulting paracrine properties like preventing apoptosis, promoting angiogenesis and tissue repair, and allowing modulation of the immune response [13,16].

Immune modulation and suppression are two essential mechanisms used through MSCs to differentiate from the cancer-associated fibroblasts (CAF) in the extracellular matrix. However, before their arrival time in the TME, certain factors in the cellular microenvironment (i.e., hypoxia and extracellular vesicles) allow the progenitor MSCs to release secretomes, enabling them to switch between them pro-inflammatory and anti-inflammatory subtypes [13]. Secretomes are soluble molecules that include proteins and peptides like cytokines, chemokines, and growth factors that favor angiogenesis and immune suppression. This activation allows the stem cell to release proangiogenic and immunosuppressive factors like vascular endothelial growth factor (VEGF), insulin growth factor-1 (IGF-1), fibroblast growth factor (FGF), hepatocyte growth factor (HGF), interleukin 6 (IL-6), prostaglandin-E2 (PGE2), indoleamine 2,3-dioxygenase (IDO), and transforming growth factor-β (TGF-β), inducing cell-cycle arrest.

As previously explained, as cancer cells escape the final stage of immunoediting, the exhaustion of T-cells causes an immunosuppressive state due to the upregulation of pro-inflammatory factors by the immune system (i.e., tumor necrosis factor-α [TNF-α] and interferon-γ [IFN-γ]). However, when MSCs encounter the TME, the downregulation of pro-inflammatory factors causes the release of suppressive cytokines and inhibitory ligands like interleukin-10 (IL-10), TGF-β, PGE2, IDO, programmed death cell receptor 1 (PD-1), and programmed death-ligand 1 (PD-L1), producing an anti-inflammatory response [26]. The resulting upregulation of anti-inflammatory cells like regulatory T-cells (Treg) releases IL-10, interleukin-35 (IL-35), and TGF-β, contributing to the inhibition and inactivation of tumor-dependent growth factors.

In addition, because MSCs can differentiate into the numerous cell types seen in the bone microenvironment, applying them to damaged bone locations can be beneficial in filling bone deficiencies in patients with OS. When undifferentiated stem cells enter the TME, their differentiation into osteoblasts causes further proliferation of osteocytes. In the paradigm, their ability to release factors to regenerate the bony matrix can be used as a physiological approach to manage and restore bone health post-surgical resection and against chemotherapeutic agents [27].

**Figure 4:**
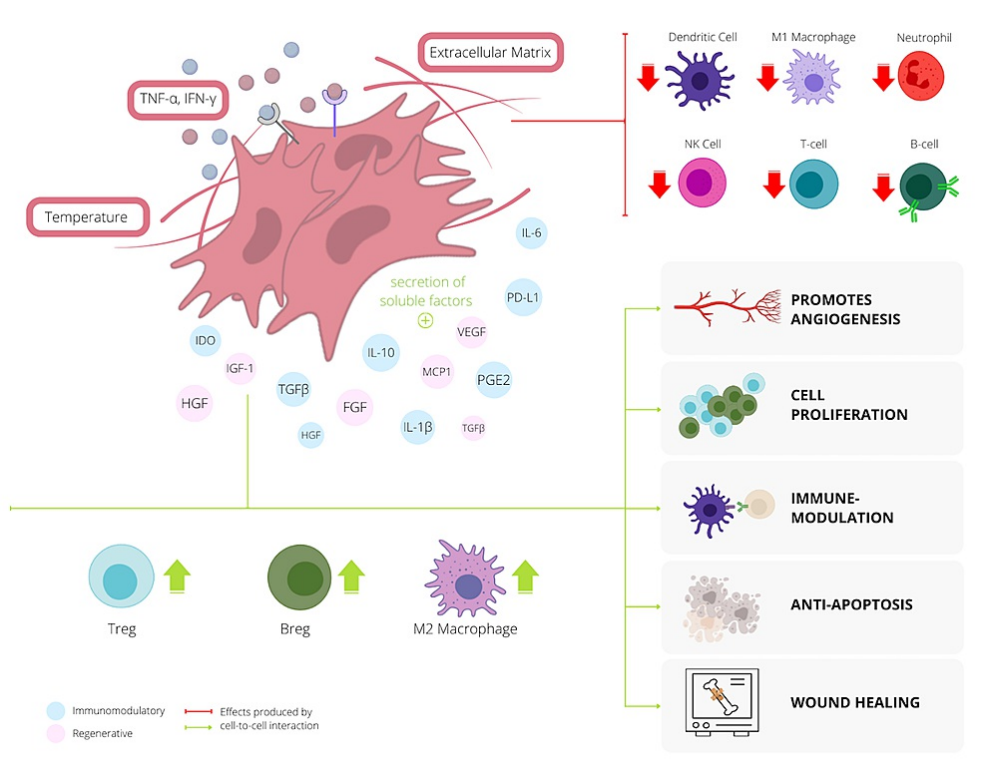
The various effects produced by cell-to-cell interaction of mesenchymal stem cells (MSCs) and the immunomodulators of the immune system. Abbreviations — Breg: regulatory B-cell; FGF: fibroblast growth factor; HGF: hepatocyte growth factor; IDO: indoleamine 2,3-dioxygenase; IGF-1: insulin growth factor-1; IL-1β: interleukin-1β; IL-10: interleukin-10; IFN-γ: interferon-γ; MCP1: monocyte chemoattractant protein-1 (also known as CCL2); NK: natural killer; PD-L1: programmed death-ligand 1; PGE2: prostaglandin-E2; TGF-β: transforming growth factor-β; TNF-α: tumor necrosis factor-α; Treg: regulatory T-cell; VEGF: vascular endothelial growth factor.

Mesenchymal stem cells possess uniquely exploitable properties related to the resolution of inflammation, tissue repair, and regeneration [13]. Their ability to mature into various immune cells in-vivo is essential for preventing autoimmunity and maintaining immune tolerance. Overall, since MSCs carry the ability to activate or inhibit the immune system response by promoting inflammation during underperformance or suppression when the immune system is overactive [16], stem cell therapy for metastatic and refractory OS introduces a promising modality for regenerative medicine and antitumor therapy in children. Figure 4 below outlines the feedback and latter effects delivered by MSCs when certain factors of the cellular microenvironment stimulate the release of secretomes.

Immune checkpoint inhibitors and osteosarcoma

Immunotherapies have become the forefront of anticancer therapy with a promising approach using immune checkpoint inhibitors (ICI). Checkpoints are inhibitory proteins expressed on immune cells, cancer cells, and other supporting cells of the TME, which assure that healthy cells are not destroyed falsely during an immune response. However, cancer cells may escape the immunological checkpoints, overall avoiding identification and destruction by the T-cells. To counteract this, inhibiting checkpoint proteins from binding to their partner protein using monoclonal antibodies may improve T-cell ability to destroy cancer cells.

Considering the expression of checkpoints varies among many cancer patients, including those with unique subtypes; cytotoxic T-lymphocyte-associated protein 4 (CTLA-4), PD-1, receptor activator of nuclear factor kappa-B ligand (RANK-L), GD-2, IGF-1, TNF-related apoptosis-inducing ligand (TRAIL), and VEGF are potential targets in ICI therapy in patients with OS. PD-1 is a transmembrane surface protein found on activated T-cells that bind to its ligand, PD-L1, expressed on the surface of antigen-presenting cells and tumor cells. The binding of the ligand results in an inhibitory signal causing inactivation of the T-cell [14,28]. Likewise, cytotoxic T-lymphocyte-associated protein 4 (CTLA-4) contributes to the down-regulation of the cytotoxic-T cell response by inducing apoptosis of neoplastic cells by binding to its ligand B7-1/B7-2 [29]. The combination of blocking PD-L1 and CTLA-4 has been encouraging in previous trials, demonstrating response to the metastatic OS and promoting immunity for progression-free disease (PFS) [30-31].

Pembrolizumab and nivolumab are anti-PD-1 antibodies employed as a potential immunotherapy for OS. In the advanced bone sarcoma arm of the SARC028 trial using pembrolizumab, 2/40 (5%) evaluable patients achieved partial response (PR) to therapy, and 8/39 achieved PFS with little to no PD-L1 expression [32-33]. However, patients in a recently reported phase II trial of pembrolizumab with recurrent and advanced OS did not achieve disease control after 18 weeks of treatment. The report suggests general resistance targeting PD-1/PD-L1 in children and that the expression of PD-L1 may not be sufficient for the antitumor activity of ICIs targeting PD-1. Per the trial, the lack of response to treatment against PD-1 in OS can be partially due to the increase in TGF-β and Wnt signaling in the immunosuppressed TME [34].

In another recently updated PD-1 inhibitor trial from National Cancer Institute (NCT02500797), nivolumab is used with and without ipilimumab (targeting CTLA-4); nivolumab prevented disease progression in 5/11 (46%) patients with OS during the first six weeks after treatment initiation but only endured in one patient (9%) after eight weeks of treatment, with only two patients expressing PD-L1 (1-3%) [35]. Ipilimumab showed disease control (DC) in 25% of OS patients, yet at a higher dose than the maximally tolerated dose, and caused grade III colitis in one of two patients with stable disease (SD) beyond the six weeks treatment [22,31,36].

Insulin-like growth factor-1 and its receptor (IGF-1/IGF-1R) have similarly been over-expressed in OS, relating to its progression and metastatic capability. After much success during a 2015 phase I clinical trial in adults with bone sarcoma treated with figitumumab, 107 pediatric patients participated in a phase II study with encouraging results. The median overall survival time with figitumumab was 8.9 months, with 15/106 (14.2%) with partial response (PR) and 25 patients with SD. The study concluded a strong association (p = <0.001) between pre-treatment serum IGF-1 and survival benefit with an overall median of 10.4 months [37]. In more recent clinical trials, cixutumumab has shown substantial stability in patients with active disease. A combination phase II study of cixutumumab and temsirolimus for recurrent, advanced, and metastatic OS produced overall SD in a total of 98 patients and PR in an additional four patients after 12 weeks of treatment [38]. Another trial using cixutumumab as monotherapy achieved PFS in 27.3% of the OS patients [39].

Other notable checkpoint inhibitors like denosumab, dinutuximab, and bevacizumab also have promising potential in pediatric OS when combined with traditional therapy. Denosumab, a targeting agent against RANK-L employed by Children Oncology Group (NCT02470091), gave a positive response in six patients (CI: 0.667 [0.195 to 0.904]), who had complete resection of all metastatic sites one month before treatment. Dinutuximab, an anti-GD-2 antibody, was combined with sargramostim (granulocyte-macrophage colony-stimulating factor [GM-CSF]) by National Cancer Institute (NCT02484443) and provided DC in 11/39 (28.2%) patients with recurrent and metastatic OS. Additionally, bevacizumab, a VEGF inhibitor, was combined with either cisplatin, doxorubicin, and high-dose methotrexate, or ifosfamide, etoposide, and high-dose methotrexate, to compare the effects in localized-resectable and metastatic-unresectable OS. The three-year event survival rate by the Kaplan-Meier (K-M) method included 31 of 43 patients (0.575 [0.402 to 0.747]) with a positive response. Despite the premature end of this clinical trial, 32/40 (76%) patients had good histological response 10 weeks after initiation of therapy with 50% or less of the tumor viable, concluding its use could provide substantial improvements in adjunctive pharmaceutical dosing [40].

Though the successful application of immunotherapy targeting specific receptors has shown to progress survival in various cancers, the consistency of poorly responsive disease to monotherapy in children could be due to low OS immunogenicity and poor localization of expressive genes. The above results suggest that combining traditional therapy with ICIs may improve immunotherapy success in the future. However, further investigation of expressive receptors in OS can improve our potential to develop new and effective targeted treatment options for children with advanced, metastatic, and unresectable osteosarcoma. A summary of the results from various clinical trials employing ICIs for specific molecular targets in OS can be found below in Table 2.

**Table 2:** Latest immunotherapy trials utilizing immune checkpoint inhibitors in patients with recurrent, relapsed, refractory, or metastatic OS <18 years of age, as of July 21, 2021. Note: all clinical trials included were designed as open-label studies and are taking place in the United States. Abbreviations – ADA: anti-drug antibody; AE: adverse events; CBR: clinical benefit rate; cis: cisplatin; Cmax: maximum plasma concentration; CR: confirmed response; DC: disease control; DLT: dose-limiting toxicity; DoR: duration of response; dox: doxorubicin; DR: disease response; EFS: event-free survival; Exp.: expression; GM-CSF: granulocyte-macrophage colony-stimulating factor; GPNMB: transmembrane glycoprotein NMB; HR: histological response; ifos: ifosfamide; IGF-1R: insulin-like growth factor 1 receptor; ir-RC: immune related response criteria; LRD: localized resectable disease; MAP: high-dose methotrexate; MD: metastatic disease; mo(s): months; MTD: maximum tolerated dose; no.: number; NCI: National Cancer Institute; NR: not recruiting; OMS: overall median survival; ORR: objective response rate; OS: overall survival; PET: positron emission tomography; PD: progression of disease; PFS: progression-free survival; Ph: phase; PK: pharmacokinetics; PR: partial response; pt(s): patient(s); RECIST: response evaluation criteria in solid tumors; SD: stable disease; t1/2: half-life; TRR: tumor response rate; UTAI: up to and including; w/: with; w/i: within; wk(s): week(s); yrs: years.

Author(s) (NCI trial No.)	Study design	Ph	Status	Drug(s) used	Primary outcome	Secondary outcome	No. of OS pts <18 years	Outcome(s)
Lussier et al. [30] and Merchant et al. [31] (NCT02500797)	Randomized, crossover	II	Active, NR	Nivolumab +/- ipilimumab	Pts w/ CR (UTAI 44 mo)	DoR, CBR, PFS and OS (6 mo)	N/A	CR: initial single therapy (IST) = 2/42 (4.8%) vs. initial dual therapy (IDT) = 6/42 (14.3%) DoR: IST: 7.4 mo (3.2 to 11.6), IDT: 6.2 mo (1.4 to 14.1) 6 mo CBR in IST: 10 pt (3 to 22), IDT: 12 pt (6 to 28) PFS: IST = 1.7 mo (1.4 to 4.3), IDT = 4.1 mo (2.6 to 4.7); OMS: IST = 10.7 mo, IDT = 14.3 mo
Tawbi et al. [32] and Keung et al. [33] (NCT02301039)	Non-randomized cohort	II	Active, NR	Pembrolizumab	ORR (8 wks UTAI 5 yrs)	AE, PFS, ir-RC, OS (UTAI 5 yrs)	6	Bone sarcoma arm: ORR: 5% (5.5-25.3) 9/42 with AE; PFS in 8 (7 to 9); ir-RC: PR = 2 and CR = 0; OS: 52 (40 to 72)
Juergens et al. [37] (NCT00560235)	Non-randomized cohort	I/II	Complete	Figitumumab	ORR	PFS, OS, ADA titer	107	ORR = 14.2% (8.1 to 22.3), 25 = SD PFS = 1.9 mo (1.8 to 2.1); OS = 8.9 mo (7.2 to 10.8) = modest activity as monotherapy. Strong association b/w pretreatment serum IGF-1 and survival benefit identified (median 10.4 mo = p <0.001)
Schwartz et al. [38] (NCT01016015)	Non-randomized cohort	II	Complete	Cixutumumab + temsirolimus	RECIST 1.1 PFS (12 wks)	—	4	PFS = PR = 4, SD = 98, PD = 56
Asmane et al. [39] (NCT00668148)	Non-randomized cohort	II	Complete	Cixutumumab	RECIST 1.1 PFS (12 wks)	PFS (UTAI 105.4 wks), ORR, OS, CBR	N/A youngest pt: 17 y/o	PFS = 27.3% (8.5 to 50.4), total = 31.9% (23.0 to 41.0); PFS (105.4 wk) = 6.4% (5.1 to 12.1), total = 6.7% (6.0 to 11.0); ORR: 5.6% (0.1 to 27.3), total = 1.8% (0.2 to 6.4); OS: 24.1 wks (12.6 to 37.6), total = 38.4 wks (31.1 to 52.0); CBR: 33.3% (13.3 to 59.0), total = 41.4% (32.2 to 51.2)
Turner et al. [40] (NCT00667342)	Non-randomized, parallel	II	Complete	Bevacizumab + cis + dox + MAP/bevacizumab + MAP + ifos + etoposide	Pts w/ DLT 3-yr EFS	Stratum HR, 2-yr EFS, 2-yr OS	43	Bevacizumab dosage scaling using ideal body weight would provide an improved dosing approach in children by minimizing PK variability and reducing likelihood of major wound healing complications. (p <0.05) 3-year EFS in LR: 0.575 (0.402 to 0.747), HR at wk 10: 24/42 (LRD + MD) w/ 5-50% tumor (grade IIB) seen, 11/42 w/ <5% tumor (grade III) seen, 2-yr EFS = 0.617 (0.470 to 0.764), 2-yr OS = 0.880 (0.782 to 0.978)
Children’s Oncology Group (NCT02470091)	Non-randomized cohort	I/II	Active, NR	Denosumab	DC (4 and 12 mo)	PK	42	Cohort I: measurable disease (RECIST 1.1) vs. cohort II: complete resection of all sites of MD w/i 30 d before enrolment at 12 mo, outcome in cohort II > cohort I — 6 patients w/ response (0.667 (0.195 to 0.904)
NCI (NCT02484443)	Non-randomized cohort	II	Active, NR	Dinutuximab + sargramostim (GM-CSF)	DC (12 mo)	t1/2	33	DC: 11/39 (28.2%) t1/2α = 0.8 (0.566 to 1.89), t1/2β = 7.5 (7.25 to 7.86), Cmax = 18.4 (7.58 to 26.3)
NCI (NCT00831844)	Non-randomized cohort	II	Complete	Cixutumumab (anti-IGF-1R)	DR	—	8	DR > 24 wks = 0/9 pts
Kopp et al. [41] (NCT02487979)	Non-randomized cohort	I	Active, NR	Glembatumumab (CDX-011 against GPNMB)	Pts w/ DLT, 3-yr EFS	HR	Median age: 20.09	DC in 3/22 (13.6%), 1/22 = PR, 2/22 = SD (extent of DC was not met for stage II), glycoprotein NMB (GPNMB) exp.: 13/19 with 3+ staining (strong) = no relation to Exp./response
Merchant et al. [42] (NCT00428272)	Non-randomized, parallel	I	Terminated	Lexatumumab (HGS-ERT2) +/- ifn-γ	MTD/DLTs (6 mo), PK (2 yr)	TRR, Exp. of pro-apoptotic proteins, ADA titer	N/A	TRR (3-24 cycles): SD = 5, CR/PR = 0, evidence of anti-tumor activity: 1 pt with recurrent progressive OS experienced resolution of clinical symptoms, PET activity and SD > 1 yr

Adoptive cell transfer and osteosarcoma

T-Cell Receptor Immunotherapy

Adoptive cell transfer (ACT) is a newly understood technique used to elicit immune responses in patients with weakened immune systems. The engineered method used by immunologists generally involves harvesting immune cells from a patient or healthy donor, modulating it ex vivo, and reinfusing it into the desired patient [29]. The reinfused donor immune cells migrate toward the tumor site and mediate antitumor effects [30]. Since T-cells play a vital role in navigating tumor-specific immune responses, the potential to use adoptive T cell transfer (ATCT) may provide a revolutionary approach when treating solid tumors compared to other forms of immunotherapy.

When T cells with desired functionalities and specificities become known, they can be collected and expanded in vitro, avoiding potential adverse reactions in vivo. To enhance T lymphocyte growth ex vivo, interleukin-2 (IL-2) can be added without impairing the function of effector T cells [12]. While sufficient quantities of autologous T cells can be produced for subsequent infusion, the TME can also be modified, allowing cancers to become more receptive to ATCT before its administration. Strategies like blocking immunosuppressive cells (i.e., eliminating Treg cells) serve as a significant advantage for ATCT [43-44].

Though ATCT strategies have been successful in patients with other types of cancer, tumor-infiltrating lymphocytes (TILs) and unmodified CD8+ T lymphocytes (CTLs) have not been fully implicated in treating OS. Potential limits in the effectiveness of these therapies may be owing to (1) ineffective recognition of target antigens since OS cell lines show a low frequency of neoantigen reactive T cells and (2) major histocompatibility (MHC) complex dependency to specific haplotypes like human leukocyte complex (HLA) class I [12].

In comparison to other ATCTs, adoptive γδ T cell transfer can provide potential advantages. Namely, γδ cells, which are human lymphocytes of the innate immune system, allow for the natural recognition of tumor antigens independent of MHC expression or sarcoma-specific histotypes, potentially benefiting all OS patients [45]. Kato et al. document γδ T lymphocytes with a capacity to directly identify and destroy OS cells. The effect is due to human tumor cells presenting aminobisphosphonates (NBPs) to γδ T cells, stimulating the production of IFN-γ and overall enhancing the antitumor activity [46]. In preclinical studies, the recognition of phosphoantigens like zoledronic acid, a potent NBP, increases the killing activity of γδ T lymphocytes against OS cells, suggesting thus the combination of adoptive γδ T cells transfer and IFN-γ with potential benefit for the future therapy [46-47].

Since the combination of ATCT and traditional therapy has achieved clinical response (>50%) in other cancers like metastatic melanoma, the potential to use this technology in OS can be revolutionary. However, before ATCT can be effective, precise recognition of target antigens and methods to upregulate HLA class I are crucial. Thus, other forms of immunotherapy with ATCT that enhance these characteristics may offer a new approach toward anti-OS activity in the future, overall justifying the further need for clinical trials in children with OS.

Chimeric Antigen Receptor T-Cell Immunotherapy

Chimeric antigen receptor T (CAR-T) cell therapy is on an uprise in treating aggressive pediatric cancers, questioning its use in childhood OS. CAR-T immunotherapy offers an adoptive therapy that uses gene-transfer technology to engineer traditional T-lymphocytes into conventional T cells [8]. The primary goal is to adjust the patient's DNA by introducing the gene coding for chimeric antigen receptor (CAR), rendering them specifically to eliminate cancer cells without needing an MHC [20]. This feature serves essential since a major component causing tumor progression in OS is the decrease in MHC class I expression [17].

The CAR genes include three significant domains: ectodomain, a transmembrane domain, and an endodomain. The ectodomain comprises a single peptide, an antigen recognition region, and a spacer exposed to the extracellular space [48-49]. The antigen recognition region is a single-chain variable fragment (scFv) composed of heavy and light monoclonal antibodies that target the selected tumor antigen (i.e., CD19 for acute lymphoblastic leukemia). The spacer connects the antigen-binding region and the transmembrane domain [50]. This domain provides stability to the receptor via the hinge derived from CD8 or immunoglobulin (Ig4) molecules, their most crucial component. Finally, the endodomain is responsible for activating the T cells once CAR binds to the target antigen, thereby allowing for the intracellular T-cell receptor (TCR) signaling [8,20].

Based on the overall transformational structure of the domains, CAR-T cells can be divided into approximately four generations. While designing each generation of CAR-T cells, careful selection of the target antigen is imperative. The first generation contains the scFv and the activating portion (CD3ζ) for TCR signaling. The following two domains include adding at least one co-stimulating domain (i.e., CD27, CD28, CD134, CD137). The last generation of CARs adds interleukin-12 (IL-12) [21], a crucial pro-inflammatory cytokine secreted by macrophages to induce differentiation of T cells and activate NK cells.

Human epidermal growth factor (HER2) is a tumor antigen highly expressed in pediatric solid tumors, including medulloblastoma (MB), OS, nephroblastoma, and rhabdomyosarcoma (RMS). In a phase one clinical trial from Baylor College of Medicine (NCT00902044) of 19 patients (median age = 14) with advanced HER2-CD28+ sarcomas, the administration of lymphodepletion (LD) chemotherapy was given to decrease the number of current T-cells and allowing room for new CAR-T cells, followed by autologous HER2-CAR-T cells, which was safely tolerated, providing SD in 3/16 OS patients past 12 weeks of therapy [51]. Impressively, one child with metastatic OS to the lungs had a complete response (CR) for 35 months; however, five days of supportive care for eight patients who experienced treatment-related cytokine release syndrome within 24 hours of receiving CAR-T cells was required [52]. Nonetheless, the trial shows good association with objective clinical benefit in patients with advanced HER2+ sarcoma.

Another highly expressed antigen, GD2, has been suggested as a potential target for CAR-T cell therapy due to restricted expression in healthy tissue versus cancerous tissue. The disaloganglioside implicated in signal transduction, cancer cell proliferation, and migration [53], most recently completed a phase I clinical trial (NCT02107963) on GD2+ solid tumors to determine the safety of administering escalating doses of a new third-generation anti-GD2-CAR-T cell. The study included a vector as an additional safety measure, caspase dimerization domain (ICD9), to induce autolysis if toxicity occurs. Cyclophosphamide-based LD and AP1903, a dimerizing agent, is also executed to enhance the clearance of anti-GD2-CAR-T cells facing toxicity. Several investigations reported that patients with ganglioside GD2 expression had a considerably shorter median survival time of the tumor than patients who did not [53-55]. The full potential to target GD2, however, is yet to be determined.

Although in its early stages of clinical research, CAR-T cell therapy is being safe and well-tolerated in patients, with little to no adverse effects. Notwithstanding the trials discussed above, there are nine other registered clinical trials implementing CAR-T technology in children. Hopefully, future clinical trials using the combination of CAR-T therapy will improve the outcomes in patients experiencing an unfavorable prognosis when treated with conventional therapies alone.

NK Cell-Based Immunotherapy

Natural killer (NK) cells are lymphocytes in the innate immune system that actively recognize targets without specific antigens [15]. In the peripheral blood, spleen, and lymph nodes, NK cells play a crucial role as the first-line defense for tumor elimination, delivering cytotoxic effects, producing cytokines, and sequentially eliminating tumor cells [56]. The primary cytokines activated by NK cells include TNF-α, IFN-γ, GM-CSF, and chemokine ligands. In the recently published meta-analysis, Zhang et al. conclude that chemokine ligands CCL5, CCL8, CCR4, and CCR5 are potential prognostic markers indicating prognosis in childhood OS [19]. Though solid tumors often have poor NK-cell colonization due to many inhibitory signals, a higher infiltration of NK cells is related to a more favorable prognosis [15]. As a result, targeting an inhibitory signal could provide a valuable strategy for restoring NK cell cytotoxicity against cancer cells.

The dual variations of NK cell immunotherapy represent a new approach for pediatric patients with OS and other solid tumor malignancies (Table 3). The first type incorporates direct targeting of cytokines and receptors involved in NK cell proliferation and function. Two of the most widely used cytokines to target NK cells are IL-2 and interleukin-15 (IL-15). However, the use of IL-2 to create lymphokine-activated killer (LAK) cells yielded mediocre results, primarily attributable to their simultaneous development of Treg cells.

**Table 3:** A summary of ongoing clinical trials for osteosarcoma using NK cell therapy. Abbreviations – CNS: central nervous system; EWS: Ewing sarcoma; MM: multiple myeloma; NB: neuroblastoma, NCT: National Clinical Trial; OS: osteosarcoma; RMS: rhabdomyosarcoma; STS: soft tissue sarcoma.

NCT trial number	Phase	Status	Start year	Title	Type of sarcoma	Country
NCT02100891	II	Active, not recruiting	2014	Phase 2 STIR Trial: Haploidentical Transplant and Donor Natural Killer Cells for Solid Tumors (STIR)	EWS + NB + RMS + OS + CNS tumors	USA
NCT02409576	I/II	Recruiting	2015	Pilot Study of Expanded, Activated Haploidentical Natural Killer Cell Infusions for Sarcomas (NKEXPSARC)	EW + RMS + OS	Singapore
NCT01807468	II	Unknown	2013	Haploidentical Stem Cell Transplantation and NK Cell Therapy in Patients With High-risk Solid Tumors	NB + EWS + RMS + OS + STS	Korea
NCT02890758	I	Recruiting	2015	Phase I Trial of Universal Donor NK Cell Therapy in Combination With ALT803	STS + EWS + RMS + OS + lymphomas + leukemia + MM	USA

In comparison to IL-2, IL-15 is far more effective at targeting NK cells for tumor therapy. Tumor cells treated with IL-15 show an expansion of NK cells and CD8-effector memory T-cells [57-58], killing cancer cells via stimulating the release of perforin and granzymes. Other cytokines, such as IL-12, interleukin-18 (IL-18), and interleukin-21 (IL-21), have also been shown to enhance the functioning of NK cells attempting to destroy chemotherapy-resistant OS cells [57,59].

**Figure 5:**
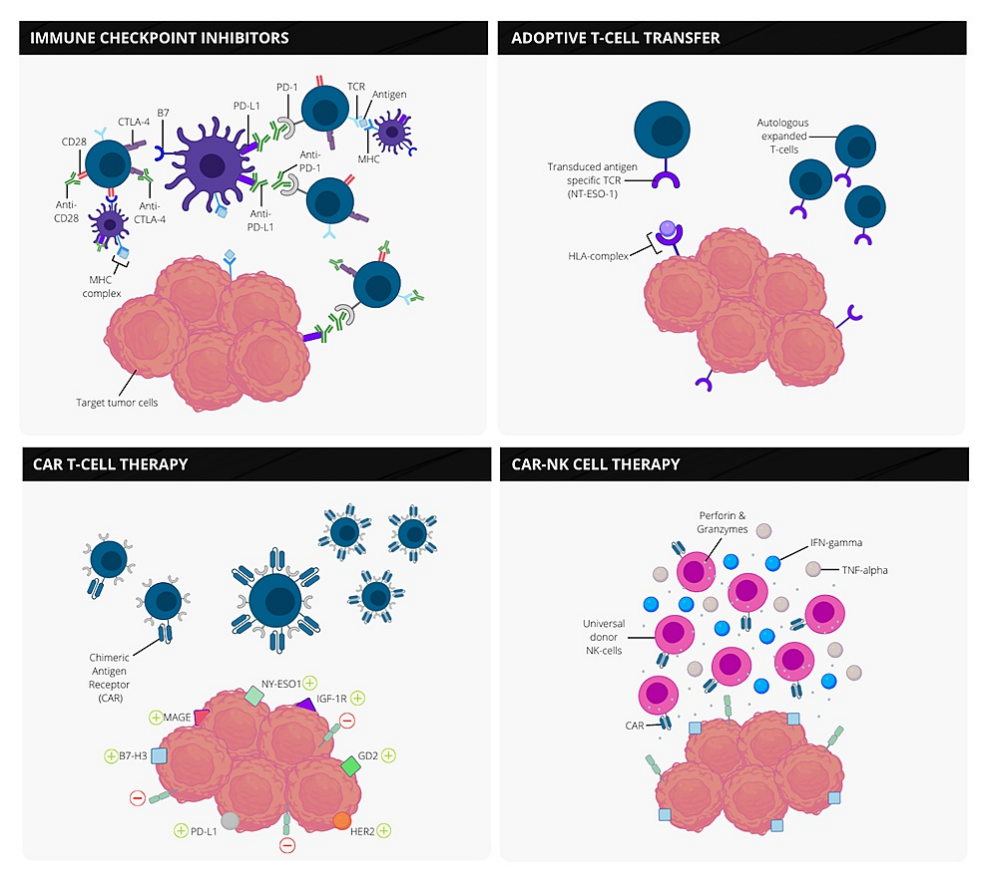
The mechanisms of various immunotherapies for the potential use in pediatric osteosarcoma. (1) ICIs target tumor cells by preventing the binding of receptors to their ligands using monoclonal antibodies. (2) ATCT involves expanding antigen-specific T cell receptors that bind to specific receptors based on the genetic makeup of the patient's tumor. CAR-T and CAR-NK cell therapy involves specific gene transfer technology to enhance the tumoricidal ability of traditional T cells by recognizing various antigens expressed by cancer. Similarly, NK cell therapy without CAR causes an increased release of perforin and granzymes to destroy unwanted cancer cells. Abbreviations* *– CTLA-4: cytotoxic T-lymphocyte associated protein 4; PD-L1: programmed death-ligand 1; PD-1: programmed cell death protein 1; TCR: T-cell receptor; MHC: major histocompatibility complex; NY-ESO-1: New York esophageal squamous cell carcinoma 1; HLA: human leukocyte complex; IGF-1R: insulin-like growth factor 1 receptor; MAGE: melanoma antigen gene protein; HER2: human epidermal growth factor receptor 2; ICI: immune checkpoint inhibitor; ATCT: adoptive T cell transfer; CAR-T: chimeric antigen receptor T; NK: natural killer.

The second class of NK-cell-based immunotherapy introduces CAR-engineered NK cells (Figure 5). Much like CAR-T cells, an intracellular signaling domain and a costimulatory signaling domain are the basic structures for a CAR-NK [50]. Other ectodomain molecules like DNAX-activation protein (DAP) 12, DAP10, NKG2D, or antigens like HER2 and GD2 can also be selected. Treatment with CAR-NK cells provides boosted tumoricidal capacity with the added benefit of not causing graft-versus-host disease or causing cytokine storms. Nevertheless, the ex vivo growth of primary NK cells remains the most challenging aspect of this treatment [60].

While some clinical trials are in progress to study the therapeutic effects of NK cell therapy, a pilot study conducted for pediatric patients with refractory solid tumors given haploidentical NK-engineered cells led to 50% survival at 14 months, resulting in partial and complete responses [61]. A pediatric patient in the same trial with RMS had resolution of lung metastases following NK stem cell therapy [13]. Another trial (NCT03209869) using IL-2 expanded in autologous NK cells was unfortunately suspended early due to limited resources from the coronavirus disease 2019 (COVID-19) pandemic.

To summarize, using surface receptors and involved cytokines to unleash NK cell antitumor responses could lead to beneficial immunotherapeutic treatments for OS. Furthermore, the effectiveness of NK cells to express CARs is noteworthy. However, more extensive studies estimating the actual effect of NK therapy in children are highly needed. Even though NK cell therapy faces significant challenges, its impressive results in various malignancies make it a viable new treatment option. Figure 5 highlights the mechanisms of the various methods of immune therapy discussed, followed by Table 3, which lists the most recent clinical trials being employed for NK cell therapy in pediatric OS.

Limitations

The following factors may have limited this study to an extent:

1. Our analysis sample size is relatively small, relying on two databases, PubMed and PubMed Central, which may interfere with the actual quality of the primary studies involved.

2. The heterogeneity of the inclusion criteria limited the potential identification of studies related to our scope of the topic.

3. The data from the current study contain clinical trials with age samples included that were beyond our inclusion criteria, studies with low pediatric patient enrolment, ongoing trials where effects have not yet been analyzed, and the lack of comparative, randomized clinical trials. Thus, providing evidence that more extensive clinical trials are required to improve the quality and reliability for future studies when assessing therapy outcomes in children with osteosarcoma.

## Conclusions

In its frontier of therapy, immunotherapy has become a promising treatment option for patients displaying receptor-positive malignancies. Treatments aim to reestablish the immune system's capacity to recognize cancer cells and effectively destroy them by overcoming the immune responses delivered by tumor cells. In general, immunotherapies have been related to less toxic effects than chemotherapy, yielding a significant appeal to treat children with cancer.

However, since the immune systems of children and adults differ significantly, a more detailed understanding of specific mutations and the genetic makeup driving osteosarcoma is necessary to fully assess the future implications of immunotherapies and their related toxicities in children. Individual factors like HLA genotypes, sarcoma subtypes, and biomarkers expression are some of the most challenging features to record while understanding the intricacy of the tumor.

This review summarized the preclinical and clinical research conducted thus far on the potential immunotherapies for children with osteosarcoma, including a particular focus on the innate and adaptive immune responses which contribute to the tumor microenvironment, tumor progression, and metastasis. An in-depth analysis of potential therapeutic pathways against childhood OS was explored, including the most up-to-date results in clinical trials utilizing immune checkpoint inhibitors and forms of adoptive cell therapy.

Even though immunotherapies have revolutionized the clinical world of oncology since their original introduction, their results against recurrent, refractory, and metastatic osteosarcoma in children are relatively mediocre. Nonetheless, it appears that combination therapies, remarkably immune checkpoint inhibitors integrated into the current standard of therapy, carry the most promising approach for children with osteosarcoma moving forward. Furthermore, while the final contribution of immunotherapy in the outcome of childhood osteosarcoma is still in its early phases, the landscape of therapy is hopefully expected to be very different from standard surgery, radiation, and chemotherapy.
